# Crisis and risk management in poultry production: Preparing future leaders for sustainability and food security

**DOI:** 10.1016/j.psj.2025.105903

**Published:** 2025-09-26

**Authors:** Farid S. Nassar, Ahmed O. Abbas

**Affiliations:** Department of Animal and Fish Production, College of Agricultural and Food Sciences, King Faisal University, Al-Ahsa, Saudi Arabia

**Keywords:** Animal Protein, Poultry Production, Crisis and Risk Management, Food Security, Sustainability

## Abstract

Poultry production sector is a cornerstone of food security at national and global levels, providing a vital source of animal protein. However, it faces mounting challenges, including epidemics, avian diseases, supply chain disruptions, climate change impacts, and rising production costs. Despite the significance of these risks, crisis and risk management remain underrepresented in higher education curricula for this essential industry. This study explores the importance of developing the knowledge and skills of poultry science students in crisis and risk management as future leaders in the poultry industry, as well as enhancing their employability, with the aim of ensuring sector sustainability and strengthening food security. A qualitative descriptive methodology, including an extensive literature review, was employed to assess the impact of crises and risk management on industry sustainability and on shaping student competencies for effective leadership in addressing challenges. Findings reveal a significant gap in poultry science education regarding crisis and risk management, undermining graduates’ readiness to lead during real-world emergencies. Simultaneously, stakeholders and academic institutions increasingly call for embedding these essential skills into curricula, recognizing their role in enhancing graduate employability and industry resilience. The study recommends reviewing and developing poultry science programs to incorporate concepts of crisis and risk management, alongside providing advanced training in collaboration with industry partners and stakeholders. It also emphasizes the need to recognize risk management as a core component of quality assurance standards within poultry science programs to ensure sector sustainability and promote academic and professional excellence. Additionally, the study provides guidance to policymakers and decision-makers in higher education institutions and poultry science programs on the importance of preparing students to become strategic leaders capable of effectively managing crises, making informed decisions, and safeguarding the sustainability of the poultry industry in the face of future challenges with confidence and competence.

## Introduction

In today’s deeply interconnected and rapidly globalizing economy, complexity, uncertainty, and volatility are on the rise, placing unprecedented pressure on businesses and making them increasingly vulnerable to disruptive and unpredictable events. These challenges include intense market competition, macroeconomic fluctuations, shifting consumer behaviors and preferences, rapid technological advancements, natural and human-made disasters, the impacts of climate change, geopolitical instability such as the Russia–Ukraine ware, and global health crises like the COVID-19 pandemic. In this complex reality, some organizations resort to developing “resilience profiles” that enable them to anticipate crises, adapt to emerging conditions, reorganize their operations, and seize new opportunities to gain competitive advantages in the aftermath of both internal and external disruptions. To remain viable in today’s business environment, organizations must innovate, adopt more agile and proactive business models, and maintain constant readiness to navigate uncertainty. Dynamic capabilities are thus the key to overcoming these challenges effectively (Lisdiono et al., 2022).

On a global scale, projections indicate that the world’s population will reach approximately 9.8 billion by 2050. Accompanying this demographic growth are major socio-economic transformations, such as urban expansion, demographic shifts, and rising income levels, which are expected to double the demand for poultry products in the coming decades. In this context, the agribusiness sector bears a dual responsibility: ensuring global food security at affordable prices while adhering to sustainable food production practices. The poultry industry holds a distinct competitive advantage in this regard, thanks to chickens’ high efficiency in converting energy and nutrients into edible products. Furthermore, poultry production plays a pivotal role in bolstering local food security and reducing poverty, particularly in regions where alternative sources of nutrient-dense food are scarce (Kleyn and Ciacciariello, 2021).

Given this strategic importance, the poultry industry, like all other sectors, must adopt proactive crisis preparedness strategies. The degree of readiness in crisis management serves as a critical indicator of an organizations or sector’s ability to respond effectively to emergencies, especially in terms of communication and decision-making. Among the most prominent threats to this sector are foodborne illnesses such as Salmonella, which, in the event of an outbreak, can cause severe economic repercussions and erode consumer trust, alongside the ongoing risks of Avian Influenza Viruses (AIVs), which poses a serious threat to the industry’s stability and security worldwide (Ulmer, 2012).

During times of crisis, leadership plays a decisive role in unifying strategic responses and motivating teams to perform at their best. In this regard, knowledge management emerges as a foundational element, providing the necessary infrastructure for rapid yet well-informed decision-making. It also serves as an organizational memory, enabling institutions to store and leverage lessons learned from past crises to enhance resilience and responsiveness in the future (Buhagiar and Anand, 2021).

Risk management itself has evolved from being a purely technical concern to becoming a matter of strategic administrative oversight and control (Soin, 2013). While the labor market increasingly demands professionals capable of performing Enterprise Risk Management (ERM) functions, inexperienced practitioners may struggle to assess risks accurately (Huang et al., 2011). Consequently, researchers emphasize the vital role of universities in equipping students with the skills employers require, including the ability to adopt a comprehensive and global perspective on organizational operations (Webb and Chaffer, 2016; Barbosa et al., 2022).

Against this backdrop, the present study underscores the urgent need to prepare future leaders in poultry production through the integration of comprehensive educational and training activities within poultry science programs. These initiatives aim to familiarize students with the nature of crises and risks, while equipping them with the preventive and managerial skills necessary to ensure sustainable production and strengthen food security. The study is guided by four main research questions, seeking to establish a scientific and practical framework to enable this vital sector to meet both current challenges and future demands:

**Q1-** What is the concept of crisis and risk management in poultry industry?
**Q2-** What are the main types of crises and risks facing the poultry industry globally and locally, and how do they impact food security?
**Q3-** What is the importance of enhancing leadership skills in poultry science students for crisis and risk management, employability, and industry sustainability?
**Q4-** How can universities adopt effective strategies to prepare students as leaders in risk and crisis management within the poultry industry?

The poultry production sector is a cornerstone of food security at both national and global levels, providing a vital source of animal protein. However, this sector faces significant challenges in the form of a wide range of risks and crises that threaten its sustainability and food security, with impacts that are particularly severe on developing countries and their fragile economies. In light of these circumstances, there is an urgent need to prepare a new generation of leaders in poultry production who are capable of confronting these challenges effectively and efficiently.

One of the most effective approaches is the integration of comprehensive educational and training activities within poultry science programs. These initiatives aim to familiarize students with the nature of crises and risks while equipping them with the preventive and management skills necessary to ensure sustainable production and enhance food security. In this context, the study seeks to provide a thorough analysis of how poultry science programs can prepare students to become effective leaders in managing industry-related crises and risks, thereby contributing to sector development, ensuring its continuity, and enhancing graduates’ employability.

The study also addresses a clear gap in current poultry science curricula regarding crisis and risk management, which affects graduates’ readiness to lead in real-world emergency situations. Furthermore, the study offers guidance to policymakers and decision-makers in higher education institutions, poultry science programs, and related academic fields, emphasizing the importance of incorporating specialized courses and training programs in crisis and risk management. Finally, the study highlights the crucial role of these programs in preparing graduates to become strategic leaders capable of managing crises effectively, making informed decisions, and safeguarding the sustainability of the poultry industry, thereby ensuring that future challenges are met with skill, confidence, and resilience.

## Material and methods

This study adopts a qualitative descriptive research methodology to explore the importance of developing the knowledge and skills of poultry science students in crisis and risk management as future leaders in the poultry industry, as well as enhancing their employability, with the aim of ensuring sector sustainability and strengthening food security. According to Neuman (2014), descriptive research is distinguished by its ability to “present a picture of the specific details of a situation, social setting, or relationship” and “begins with a well-defined issue or question and endeavors to describe it accurately”*,* while it also “…concentrates on ‘how’ and ‘who’ inquiries…”*.* Moreover, Lambert and Lambert (2012) argue that qualitative research designs, such as phenomenology and grounded theory, can serve both descriptive and explanatory purposes. They emphasize that using the term "qualitative descriptive research" provides a more accurate characterization of such studies rather than associating them with other methodologies (e.g., phenomenology, grounded theory, ethnography). They further note that qualitative descriptive research “…often derives from naturalistic inquiry”*,* which is committed to examining phenomena in their natural state as much as possible within the research context. Given its qualitative nature, this study aims to explore the importance of developing the knowledge and skills of poultry science students in crisis and risk management as future leaders in the poultry industry, as well as enhancing their employability, thereby ensuring sector sustainability and strengthening food security, which clearly positions it within the scope of qualitative descriptive research. In line with the study’s objectives, a comprehensive content analysis of both electronic and printed materials relevant to the events under investigation was conducted (Bowen, 2009). According to Bowen (2009), content analysis comprises three essential phases: skimming, thorough reading, and interpretation. This method enables the systematic categorization of textual data into smaller, meaningful, and manageable units (Weber, 1990), with the aim of uncovering underlying meanings by identifying recurring themes and patterns within the sources (Patton, 2002).

The methodology adopted in this study followed a structured, four-phase approach. In the first phase, a comprehensive systematic search was conducted across leading academic databases, including Google Scholar, Scopus, and Web of Science, using a targeted set of keywords such as “Crisis Management,” “Risk Management,” “Poultry Production,” “Student Leadership Development,” and “Labor Market.” The search focused on peer-reviewed articles, institutional reports, and empirical studies published in English over the past five years, supplemented by foundational literature to establish a robust theoretical framework. The second phase involved defining precise inclusion and exclusion criteria. Studies were included if they directly addressed crises and risks related to the poultry industry, various concepts of crisis and risk management, and the development of students’ leadership capacities with the aim of cultivating practical skills that enhance their employability in production-oriented environments such as factories and livestock farms. Studies were excluded if they lacked a clear connection to higher education or production settings, or if they were methodologically weak or lacked empirical evidence.

In the third phase, data were extracted and analyzed from the selected sources, focusing on each study’s main objectives, key findings related to crisis and risk management, the poultry industry, and the development of students’ leadership skills in higher education institutions, as well as the identified challenges and proposed recommendations. A qualitative synthesis of the data was conducted to reveal the core benefits of enhancing students’ capacities as leaders in crisis and risk management, and to determine their strategic contribution to improving the quality of poultry science programs and fostering students’ leadership competencies within the poultry production sector. The fourth and final phase comprised an in-depth analysis of the findings to explore effective strategies for preparing students as leaders in crisis and risk management within an integrated educational framework. This phase aimed to highlight the activities and training programs that higher education institutions and poultry science programs should adopt to strengthen students’ leadership capabilities in managing crises and risks. The ultimate goal is to empower the next generation of leaders capable of addressing the ongoing challenges facing the poultry industry and ensuring its optimal sustainability.

This integrated methodological framework was designed to provide a rigorous and comprehensive assessment of the importance of preparing a new generation of poultry industry leaders equipped with advanced skills in crisis and risk management skills that will enhance their ability to meet challenges and secure the long-term sustainability of the sector.

## Results and discussion

### The concept of crisis and risk management in poultry industry

Crises and risks are an integral part of the business and production environment, including the poultry sector, where any sudden event or unexpected change can threaten the continuity of operations and the stability of organizations operating in this vital field. This underscores the importance of understanding the concepts of crises and risk management, not only to respond swiftly when they occur but also to develop preventive and proactive strategies aimed at mitigating their negative impacts on poultry production.

#### The concept of crises

Understanding how to lead effectively during a crisis is a complex challenge that first requires a clear grasp of what constitutes a crisis. This task is complicated by the diverse causes and consequences crises may have, as exemplified by the COVID-19 pandemic, which highlights the difficulty in precisely defining an event as a crisis. The term "crisis" refers to a situation characterized by difficulty and danger, threatening an organization’s core operations or survival (Hermann, 1963). Crises are marked by their rarity and unpredictability (Weick, 1988), yet when they occur, they have profound impacts that can jeopardize the very existence or fundamental functions of the organization (Hermann, 1963; Weick, 1988). These significant effects generate intense time pressure, as delayed or imprudent responses may exacerbate the damage and potentially lead to organizational collapse. Early crisis research primarily treated crises as isolated events to be prevented and contained to safeguard organizational operations and reputation, such as security breaches or product recalls (Wooten and James, 2008). However, as societies and organizations evolved, becoming more complex and interconnected, the nature of crises transformed significantly (Riggio and Newstead, 2023).

Initially, crises were conceptualized as unexpected threats imposing urgent time constraints on organizations (Hermann, 1963). Over time, this understanding has evolved considerably. A pivotal contribution came from Weick’s (1988) work on sensemaking during crises, which explained how individuals interpret raw stimuli and how their decisions and actions influence crisis development. This perspective reveals that crises are not merely objective events but are shaped and reshaped by the responses of those attempting to understand and manage them. Furthermore, Pearson and Clair (1998) emphasized the inherent ambiguity of crises, noting the frequent difficulty in precisely identifying their causes, clearly delineating their effects, or determining the most appropriate response. This ambiguity stems from the novelty of crises, which often lack established processes, creating uncertainty.

The complexity of crises is further heightened by their public nature, subjecting leaders to intense scrutiny (James and Wooten, 2005). Such public oversight means leaders’ actions during crises are closely watched, and both the real and perceived impacts can spread widely. Additionally, crises tend to be emotionally charged (James and Wooten, 2005; James et al., 2011), making the ability of leaders to manage emotions during such times a critical and valuable area of study (Wu et al., 2021). In this context, organizational capacity to respond proactively to the ambiguity surrounding crises depends heavily on managers’ situational awareness of both internal and external influencing factors, as well as on the mechanisms the organization employs to gather, analyze, and share information (Burnard et al., 2018). Moreover, effective knowledge management enhances an organization’s preparedness to understand the nature of the crisis more deeply while positioning itself strategically to develop competitive advantages during turbulent periods (Cania and Korsita, 2015).

#### The concept of risk management

There is considerable variation in the understanding of risk concepts across different branches of social sciences, with each branch relying on a distinct primary unit of analysis. Economists, for example, study risky behaviors at the individual level, strategists focus on group activities, and sociologists examine risk as a social phenomenon. Within each unit of analysis, there are additional sublayers. In the field of strategic management, risk is viewed as a multidisciplinary construct, with a primary emphasis on the role of risk and behavioral judgment in managerial decision-making and its impact on overall organizational performance, contrasting with the narrower concept of risk found in finance and economics (Acharyya and Brady, 2014).

The ISO 31000 standard defines risk as “the effect of uncertainty on objectives” that can include the organization’s purpose, vision, and values as well as the goals and targets articulated at different levels in the organization. They can also include the factors that are important to a particular decision (ISO, 2018). Risks are addressed through structured processes designed to increase the probability of success in complex and challenging environments (Olechowski et al., 2016). Organizations face various types of risks, including those related to regulatory compliance, business operations, workplace health and safety, and environmental and social issues (Shad et al., 2019). Risk management involves systematically identifying the various risks a company faces, evaluating their potential impact, and prioritizing them accordingly. Following this analysis, organizations formulate and implement action plans to mitigate these risks in a manner deemed acceptable and effective (Pérez-Cornejo et al., 2019). These are considered traditional risk management processes. However, organizations have recognized that the scope of risks has expanded to include multiple types that impact enterprises. As a result, they have begun adopting risk management processes in a holistic manner, not only addressing individual risks but also understanding the interactions among them (Shad et al., 2019). This approach is known as Enterprise Risk Management (ERM), which enables a robust, quantitative, and economic understanding of risks, with their impacts communicated across all levels of the organization (Paula et al., 2018). ERM can be seen as an evolution of traditional risk management with the addition of corporate risk governance (Fraser and Simkins, 2016).

The importance and adoption of ERM has increased significantly due to the COVID-19 pandemic, as ERM teams have assessed supply chains, cybersecurity risks, remote work challenges, and other issues organizations face amid pandemic-related circumstances (Davis, 2020). Therefore, it is essential for students to develop competencies related to ERM, as organizations will increasingly demand these skills (Barbosa et al., 2022).

In the poultry industry, identifying risks and mastering crisis management is of paramount importance due to the sensitive and complex nature of this sector. Sudden events such as disease outbreaks, feed price fluctuations, or climate changes can threaten production continuity and the stability of operating organizations. Therefore, a clear understanding of the concepts of crises and risk management enables leaders and managers to anticipate potential threats, assess their impacts, and implement proactive measures aimed at protecting bird health, product quality, and minimizing negative effects on production. Moreover, risk management focuses on the early identification of potential threats, such as avian disease outbreaks like avian influenza or sudden feed price volatility, evaluating these risks, and applying preventive strategies, such as strengthening biosecurity protocols or diversifying feed sources, to reduce the likelihood or impact of crises. Conversely, crisis management addresses urgent events that directly threaten production continuity, such as sudden disease outbreaks causing massive livestock losses, emphasizing rapid response, damage control, and operational recovery.

In addition, when effectively applied, risk management allows poultry organizations to anticipate crises, such as extreme weather conditions affecting bird health or feed supply disruptions, enabling proactive actions like climate control interventions or securing alternative feed suppliers to enhance operational resilience. Integrating risk management into broader frameworks, such as ERM, further strengthens quantitative and economic understanding of risks and ensures that their impacts are communicated across all organizational levels.

Ultimately, mastering risk and crisis management improves an organization’s ability to adapt to unforeseen challenges, ensures sustainable poultry production, and enhances food security at both national and global levels. By combining preventive planning with the capacity for rapid response, poultry organizations can protect poultry production, safeguard resources, maintain stability, and establish risk and crisis management as a strategic cornerstone for long-term sectoral success.

### Key crises and risks facing the poultry industry globally and locally and their impact on food security

In poultry production, risk management is critical yet faces significant challenges. It is essential to raise awareness among industry stakeholders about the diverse risks they encounter, which are categorized into five main types: production risks, market risks, financial risks, legal risks, and human resource risks (Murrja et al., 2023). Adeyonu et al. (2021) reported that poultry farmers consider production, financial, and human resource risks as the most significant challenges facing the poultry production industry, and they primarily rely on disease prevention strategies and financial management to mitigate the impact of these risks. Therefore, understanding farmers’ risk perceptions is crucial to fostering the sustainable growth and resilience of the poultry industry (Adeyonu et al., 2021). Moreover, poultry industry, both locally and globally, faces a complex set of crises and risks that exert varying degrees of impact on production efficiency and the sustainability of this sector, including the following:

#### Risk of disease outbreaks

The poultry industry is the largest global source of meat and eggs for human consumption. However, viral outbreaks in farmed flocks are a common occurrence and pose a significant concern for this industry. Mortality and morbidity rates resulting from such outbreaks can lead to substantial economic losses, with consequent adverse impacts on the global food supply chain (Ravikumar et al., 2022). Additionally, AIVs are a prominent example illustrating this risk. AIVs are highly contagious respiratory pathogens affecting birds, causing high rates of infection and mortality worldwide, which result in severe economic damage to the poultry and agricultural sectors (Graziosi et al., 2024).

The rise and recurrence of highly pathogenic avian influenza (HPAI) outbreaks in North America have serious implications for the poultry industry, which currently contributes over $40 billion annually to the United States' Gross Domestic Product. When HPAI outbreaks occur, both upstream production and downstream markets experience severe disruptions, including mass culling, supply shortages, and sharp price increases (Ukaoha, 2025). The 2014–2015 epidemic alone caused the death of more than 50 million birds and economic losses estimated at $3.3 billion, with notable effects on consumer prices and trade relations across wide regions (Seeger et al., 2021).

The impact of disease outbreaks on the poultry industry and its sustainability is highly significant, as they cause substantial economic losses due to high mortality and morbidity rates among birds, reducing productivity and increasing operational costs. These outbreaks also disrupt the stability of the food supply chain, as markets fail to meet the demand for poultry products, placing additional pressure on farmers and suppliers, which can lead to price volatility and market fluctuations. In the long term, such crises reduce the sector’s capacity for sustainable growth and weaken consumer and investor confidence, thereby threatening food and economic security linked to the poultry industry. Therefore, strengthening preventive measures, enhancing veterinary surveillance, and implementing effective risk management strategies are essential to ensure the industry’s sustainability and the continuity of production in the face of such crises.

#### Epidemic diseases and their impact on poultry production

The COVID-19 pandemic and the associated lockdowns presented unprecedented challenges to the poultry sector in Bangladesh and worldwide. Farmers faced multiple difficulties related to marketing, transportation, and securing essential inputs. These harsh conditions forced producers to adapt their crisis management practices, particularly in disease control and resource utilization, with some relying on traditional knowledge and community cooperation. The poultry sector remains a vital pillar for economic growth and protein supply; however, it continues to face persistent threats from diseases such as *Salmonella* and HPAI, which undermine industry stability and consumer confidence. Although COVID-19 does not transmit through poultry, global restrictions adversely affected trade and production, making enhanced preparedness for future crises a critical priority for the sector’s strategic sustainability (Hossain et al., 2021).

The pandemic exposed significant vulnerabilities within the current economic model of the poultry sector during crises. Consequently, the industry must shift towards a production model that prioritizes market demand over indiscriminate output, alongside improving distribution systems to ensure resilience during emergencies. Furthermore, updating educational programs is essential to bolster the sector’s readiness to face future challenges and guarantee its long-term continuity (Zaime and Ouahi, 2023).

In India, the COVID-19 outbreak fueled rumors and misinformation linking poultry meat and eggs to virus transmission to humans, resulting in a sharp decline in consumption, demand, and prices of poultry products. Pandemic-related lockdowns exacerbated the crisis by disrupting feed supply and health inputs, causing spoilage of eggs, chicks, and birds, and leading to significant economic losses due to the disruption of the poultry-based animal protein supply chain. It is estimated that the Indian poultry industry incurred losses of approximately $3.053 billion as a result of this crisis (Kolluri et al., 2021).

In this context, the impact of epidemic diseases, such as the COVID-19 pandemic, on the poultry industry and its sustainability has been profound. The pandemic and associated lockdowns created unprecedented challenges across production and distribution chains, causing significant difficulties for farmers in marketing, transportation, and securing essential inputs such as feed, vaccines, and medications. These conditions also led to spoilage of eggs, chicks, and birds, resulting in substantial economic losses and negatively affecting the stability of poultry-based food supply chains. Moreover, rumors and misinformation, as observed in India, led to a sharp decline in poultry consumption and product demand, exacerbating price volatility and undermining consumer and investor confidence in the sector. The pandemic further exposed critical vulnerabilities in the current production model, including overreliance on imported inputs and inflexible distribution systems, with particularly pronounced effects in developing countries.

Consequently, it has become imperative to restructure the poultry industry toward a more resilient production model that prioritizes actual market demand, strengthens distribution networks, and integrates comprehensive crisis and risk management practices, including enhanced health monitoring and preventive planning. Additionally, updating educational and training programs to address future challenges is essential for ensuring the sector’s sustainability, protecting production continuity, and reinforcing both national and global food security.

#### Climate change challenges and their impact on productivity and sustainability

By 2050, global demand for poultry products is expected to quadruple due to rising living standards. Simultaneously, climate change threatens the quality of feed crops and pastures, water availability, poultry health, and reproduction, posing significant challenges to production. Climate variability is projected to reduce output by tripling water consumption in poultry, increasing demand for agricultural land due to rapid production growth, and raising food security concerns especially considering that about one-third of cereal crops are used as animal feed, including poultry feed. These challenges necessitate the assessment and implementation of region-specific adaptation and mitigation measures alongside supportive policies to transition towards sustainable poultry production systems (Attia et al., 2024).

Broiler strains are characterized by rapid growth, high productivity, and excellent feed efficiency when reared under optimal environmental conditions that allow the full expression of their genetic potential. However, climate change—particularly rising ambient temperatures—undermines these ideal conditions, resulting in reduced production performance, lower feed conversion efficiency, increased mortality rates, significant economic losses, and critical threats to global food security. Global broiler meat production increased by 1.63 million metric tons in 2023/2024 compared to the previous period, and it is expected to grow by approximately 2 % in 2025, reaching a new record of 104.9 million metric tons. Heat stress causes annual economic losses estimated at $2.36 billion in the U.S. poultry industry due to decreased productivity, higher mortality, increased feed costs, and disease resistance (Abbas et al., 2025).

Poultry farming represents a vital source of income for smallholder farmers in developing countries and plays a crucial role in meeting daily protein needs through meat and egg consumption. The COVID-19 pandemic has significantly impacted the poultry production sector worldwide; however, its repercussions have been particularly severe in developing nations due to their heavy reliance on imported essential supplies such as feed, vaccines, medicines, and equipment (Attia et al., 2022).

The devastating COVID-19 pandemic exposed the fragility of global supply chains. To ensure smooth continuity of production in the post-COVID-19 era, policymakers must focus on establishing sustainable and resilient supply chains by restructuring existing supply strategies. Key barriers hindering sustainability and resilience include natural and human-made disasters, quality issues related to chicks, feed, and vaccines, poor forecasting, limited access to technology, and environmental challenges (Roy et al., 2024).

Chapot et al. (2024) noted that the poultry sector in Indonesia faces multiple challenges, including high disease prevalence, significant price volatility for production inputs and outputs, and weak biosecurity measures. Providing reliable and timely information on animal production and poultry health can assist all stakeholders in the value chain to make informed management decisions aimed at enhancing profitability and productivity while minimizing public health risks.

During crises, ensuring the stability and safety of food supplies is critical. Although Germany is a developed economy with decades of food security, recent crises have revealed vulnerabilities in the national supply chain, especially in livestock production, due to challenges faced by primary agricultural production during power outages. This underscores the urgent need to focus on strengthening production resilience to better cope with future crises (Kleingräberai and Efkenb, 2025).

In this context, poultry industry faces significant challenges that threaten its sustainability both locally and globally due to climate change, health crises, and economic disruptions. Rising temperatures and climate variability lead to the deterioration of feed and pasture quality, water shortages, and declines in bird health and fertility, resulting in reduced productivity, lower feed conversion efficiency, higher mortality rates, substantial economic losses, and critical threats to global food security. On the other hand, COVID-19 pandemic further exposed the fragility of supply chains, particularly in developing countries that rely heavily on imported essential inputs such as feed, vaccines, and medicines, causing distribution disruptions, spoilage of eggs, chicks, and birds, reduced production, and diminished consumer and investor confidence due to rumors and misinformation. In addition, the industry faces considerable volatility in input and output prices, widespread disease outbreaks, weak biosecurity measures, and insufficient data for informed decision-making. Collectively, these factors underscore that achieving sustainability in the poultry sector requires restructuring production systems to be more flexible and resilient to crises through enhanced biosecurity, risk management, improved and adaptive supply chains, and supportive policies for climate adaptation. Moreover, updating educational and training programs is a strategic factor in strengthening the preparedness of industry personnel to face future challenges, ensuring production continuity, and safeguarding food and economic security in the long term, positioning institutional sustainability as a core pillar for the continued success of the poultry industry in an increasingly complex and dynamic environment.

#### Social changes and consumer behavior

Social changes, including shifts in consumer behavior, have a direct impact on crisis management strategies. Therefore, organizations must be able to swiftly adjust their policies and approaches to address various social challenges, such as the need for a more flexible workforce, adapting to legislative changes, and managing reputational risks (Nahornyi et al., 2022). Therefore, poultry industry is significantly influenced by social changes and consumer behavior, as these factors directly affect crisis and risk management by impacting market demand and stability. Sudden shifts in consumer preferences or the spread of misinformation can trigger financial and marketing crises that threaten the sector’s productivity and profitability. Therefore, the ability to monitor and understand these changes is an essential aspect of risk management, enabling organizations to adopt proactive and flexible strategies such as adjusting supply chains, diversifying products, and improving consumer communication to ensure stable demand and minimize losses during crises. By integrating social changes and consumer behavior into crisis management plans, the poultry industry can enhance its sustainability, protect productivity, and safeguard long-term food security.

In light of all the aforementioned risks and crises, poultry industry, both globally and locally, operates within a highly dynamic and complex environment that makes it particularly vulnerable to a wide range of crises and risks, including outbreaks of epidemic diseases such as HPAI and Salmonella, feed price volatility, climate change, supply chain disruptions, and shifts in consumer demand or regulatory frameworks. These challenges threaten poultry productivity, animal health, and the stability of food supplies, directly impacting food security. The COVID-19 pandemic exposed the fragility of global supply chains and highlighted the critical importance of adaptive crisis management, including disease control strategies and financial resource management, while climate change adds further risks to productivity, product quality, and economic stability. Limited reliance on traditional technologies for detecting bacterial contamination underscores the need to adopt modern diagnostic tools, such as biosensor technologies, to enhance food safety and monitor antibiotic resistance.

In light of these complexities, higher education institutions and poultry science programs should play a pivotal role in educating students about potential crises and risks, equipping them with the theoretical knowledge and practical skills needed to manage these challenges effectively, including preventive strategies, risk management, and the development of resilient and sustainable supply chains. This approach helps cultivate a generation capable of strengthening the resilience of the poultry industry, ensuring production continuity, safeguarding national and global food security, supporting economic growth, and achieving resource sustainability in the face of future crises.

### The importance of enhancing leadership skills in poultry science students for crisis and risk management, employability, and industry sustainability

Based on the challenges facing the poultry industry at both national and global levels, it has become essential for organizations and their workforce to possess the capacity to effectively confront and manage crises. Effective leadership, alongside crisis and risk management, plays a pivotal role in enhancing the resilience of the poultry sector and enabling it to adapt to economic, social, health, and environmental changes, while also building strong and sustainable organizational structures within farms and production facilities. This dynamic environment requires the poultry sector to adopt a flexible management approach and proactive strategies for risk mitigation, including understanding the dynamics of various crises, responding swiftly to economic and social fluctuations, and strengthening institutional preparedness to face shocks.

Building on this, it becomes clear that developing leadership competencies and practical skills among students in poultry science programs serves as a key tool to improve their employability and enhance their ability to make informed decisions and manage risks effectively within the sector. Therefore, integrating leadership, crisis management, and risk management concepts into poultry science programs is of critical importance. This integration aims to prepare future leaders capable of facing challenges with confidence and competence, while strengthening the resilience of the poultry industry and improving its productivity and long-term sustainability.

In parallel, higher education institutions face increasing pressure to produce employable graduates (Pitan and Muller, 2020). They are expected to develop strategies that enable their graduates to enter the labor market under the best possible conditions and within the shortest possible time (Caballero et al., 2015). One key strategy is the implementation of academic activities aimed at enhancing students’ employability, which may include curriculum design. Indeed, employability serves as a performance indicator for some universities, measuring how effectively the institution develops graduates with work readiness skills, thereby enhancing the university’s reputation and ultimately attracting more students (Hossain et al., 2019; Barbosa et al., 2022).

To achieve this, individuals require a set of competencies to continuously and successfully advance in their careers (Borg and Scott-Young, 2020), and many of these competencies must be developed at the university to facilitate students’ transition into the workforce (Cavanagh et al., 2015). When students perceive deficiencies in certain competencies, they naturally feel less confident, viewing these gaps as barriers to employment. For example, lack of experience and the quality of degrees are among the most significant employment barriers (Beaumont et al., 2016). Studies also highlight that soft skills, problem-solving abilities, functional skills, pre-graduate work experience, and academic reputation (Chhinzer and Russo, 2018) all influence students’ employability (Barbosa et al., 2022).

The shift from an education system focused on theoretical knowledge to one centered on competency-based learning has become a necessity imposed by the contemporary labor market. However, the success of this transformation requires a comprehensive strategy that clearly defines the required competencies, provides industry-related field experiences, implements precise performance assessment mechanisms, ensures continuous training for faculty members, and establishes effective partnerships with stakeholders. In specialized programs such as poultry science, this approach becomes even more critical, as orienting curricula toward an integrated mix of technical and practical skills such as biosecurity, poultry nutrition, and supply chain management alongside leadership and managerial skills, including crisis management, communication, and decision-making, significantly enhances graduates’ employability and strengthens the sector’s long-term sustainability.

Against this backdrop, employability is defined as the extent to which prospective graduates possess a set of technical and soft skills (Hossain et al., 2019) and is considered a collection of personal attributes that enable individuals to obtain and retain employment (Tavares, 2017). Work readiness reflects graduates’ potential in terms of long-term job performance and career advancement (Caballero and Walker, 2010). Being work-ready means that graduates are prepared to enter the labor market (Spanjaard et al., 2018). Work-ready students demonstrate that they possess effective knowledge and competencies that can be applied in practical work environments (Prikshat et al., 2019).

Within this context, Barbosa et al. (2022) highlighted the significant positive impact of developing competencies related to risk management standards, along with the supporting soft skills, on students’ work readiness. They also emphasized the importance of redesigning academic curricula and course programs to focus on the development of processes, techniques, and tools embedded in risk management standards. Such emphasis plays a pivotal role in enhancing soft skills, facilitating the effective application of risk management practices, and ultimately strengthening students’ perception of their professional preparedness.

This focus on preparedness is especially important in today’s world, which has become increasingly complex due to rapid civilizational progress, socioeconomic transformations, globalization, and industrialization. The core challenge lies not in the presence of crises themselves, but in individuals’ ability to manage them effectively and responsibly. Education serves as a strategic driver of social change and development, extending beyond academic knowledge to include personality growth and the cultivation of responsibility aligned with societal needs. Moreover, quality education equips individuals with the values and skills necessary to face future challenges with resilience, making it essential for higher education institutions to provide experiential learning opportunities through programs and curricula that enhance students’ self-confidence, strengthen their ability to shape their own lives, and develop the competencies required for effective crisis and risk management.

At the same time, not every leader will face major crises, but it is likely that every leader will encounter crises of varying magnitudes during their tenure. Therefore, it is essential for leaders to have a deep understanding of the different types of crises and their common dynamics, enabling them to recognize early warning signs and respond effectively as soon as a crisis arises. During crises, leaders play crucial roles including fostering collective understanding, making thoughtful and responsible decisions, communicating broadly and prudently, coordinating resources and organizing teams, and supporting continuous learning. These skills and competencies should be considered fundamental in the processes of selecting and developing leaders (Riggio and Newstead, 2023).

As research in crisis leadership grows, it becomes increasingly necessary to focus on the distinctions and intersections between leadership and crisis management, as well as the influence of international and cultural factors, and the commitment to professional ethics in crisis leadership. Given the continued emergence of crises with increasing frequency and complexity, it becomes necessary to consistently invest in research, education, and capacity building to ensure effective leadership in facing these challenges (Riggio and Newstead, 2023).

Looking ahead, the future of agriculture demands a workforce equipped with dynamic networks, innovative approaches, and proactive risk assessment capabilities (Alston et al., 2009). To meet these requirements, twenty-first-century agricultural students must develop advanced behavioral skills that enable them to be effective collaborators, innovators, and problem-solvers capable of addressing global challenges such as climate change, food security, and urbanization (Parrella et al., 2022). Juhász and Horváth-Csikós (2021) emphasized the critical role these skills play in student success across agricultural fields, highlighting the urgent need to integrate their development into postsecondary agricultural education programs. However, educators in higher agricultural education often focus on discipline-specific technical skills, neglecting to provide sufficient opportunities for students to develop essential behavioral competencies (Hendrix and Morrison, 2018).

In this vein, graduates of poultry science programs also make a significant contribution to community development by supporting local food systems that maintain economic value within regional economies and strengthen the connection between consumers and their food sources. When consumers know their farmers, they become more aware of and invested in sustainable agricultural practices. By combining academic knowledge with practical experience, these graduates not only meet immediate industry needs but also actively engage in broader global discussions on sustainability in poultry production. As a result, they emerge as informed and proactive citizens capable of driving positive change in an increasingly interconnected world (Fanatico et al., 2025).

Furthermore, leadership skills among university students can be effectively developed through activities and programs that contribute to enhancing their leadership capacities, reducing negative emotions, and supporting holistic development. The ability to demonstrate leadership qualities across various social and professional domains is of paramount importance today, as this skill strengthens students’ capacity to assume influential leadership roles in their future social and professional fields (Uaikhanova et al., 2022). Leadership training has shown a positive impact on academic and social outcomes, with students exhibiting high levels of academic engagement, positive attitudes toward learning, and beneficial study habits. However, there was limited room for improvement in academic performance and problem-solving skills. Socially, students demonstrated good peer relationships, high self-esteem, and successful teamwork, while interpersonal and conflict resolution skills required further development (Obligado et al., 2023).

These findings reinforce the idea that leadership and crisis management are inseparable. Developing and implementing effective crisis management strategies is vital for organizations at all levels and across all sectors. Effective leadership is characterized by the ability to adapt quickly to change and inspire teams toward innovation forms the cornerstone of building a strong and sustainable organizational structure. Flexibility and the adoption of a responsive management approach become even more critical during periods of uncertainty arising from global financial crises, political conflicts, and pandemics (Koroliuk et al., 2023). From this perspective, social changes, including shifts in consumer behavior, directly influence crisis management strategies. Therefore, organizations must be capable of rapidly adjusting their policies and approaches to address multiple social challenges, such as the need for a more flexible workforce, adapting to legislative changes, and managing reputational risks (Nahornyi et al., 2022).

From the above, it is evident that there is a strong interconnection between leadership and crisis management, which constitutes a vital element for the sustainability of the poultry sector. The industry faces increasing challenges, including animal epidemics, market fluctuations, climate changes, and evolving consumer demands, making the ability to identify risks early and respond swiftly and effectively essential. Leaders in this sector play a pivotal role in ensuring production continuity through responsible and timely decision-making, resource coordination, team organization, and the promotion of continuous learning within production institutions. Moreover, developing leadership and behavioral skills among graduates of poultry science programs enables them not only to meet current industry needs but also to contribute to the sustainability of local food systems, strengthen the connections between producers and consumers, and encourage sustainable production practices. Intensive training in leadership, crisis management, and decision-making flexibility enhances graduates’ capacity to address environmental and economic challenges and ensures that poultry organizations can respond efficiently to financial crises, health emergencies, or shifts in consumer behavior, thereby improving competitiveness and achieving long-term sustainability at both local and global levels.

On the other hand, from an economic perspective, crises such as financial collapses and recessions profoundly impact crisis management within organizations, requiring swift responses to financial changes, budget reassessments, investment strategies, and development plans. Case studies illustrate how some organizations have successfully adapted to economic disruptions by adopting cost-reduction strategies, restructuring operations, and diversifying income sources. Consequently, effective crisis management requires a flexible approach capable of addressing a wide range of economic, social, and other challenges. Organizations with high resilience and the ability to reformulate their strategies have a greater chance of overcoming crises successfully (Koroliuk et al., 2023).

Within this framework, leadership capabilities play a crucial role in enhancing organizational resilience and risk management practices. Risk management practices also serve as a partial mediator in the relationship between leadership capabilities and organizational resilience, enabling success in unstable and turbulent business environments—particularly in organizations operating in developing countries (Lisdiono et al., 2022).

Leadership, therefore, is the executive power that enables a company to be resilient and capable of facing challenges. The leader initiates a forward-looking dialogue among all stakeholders within the organization (Fiksel, et al., 2015)**.** Leadership from top management teams is a critical element within the strategic management framework for enhancing organizational resilience (Schoemaker et al., 2018)**.** Additionally, leaders view risks and uncertainties as a natural part of business and prepare proactively and decisively for all conceivable future scenarios. With a capable leader, resilient companies will be able to withstand crises and flourish thereafter (Lee et al., 2013; Lisdiono et al., 2022)**.**

On the other hand, effective leadership promotes proactive risk management, which improves employees’ safety behavior (Fernández-Muniz et al., 2014). During shocks, the entire organization relies on the leader to take appropriate actions that protect everyone. Therefore, a strong leader motivates the organization to manage uncertain events effectively, enabling it to navigate difficult times and return to normal or better conditions. Proactive risk management is the process of anticipating, assessing, controlling, and transferring risk, which involves recognizing, evaluating, controlling, and redirecting risks (Qing-gui et al., 2012). Risk management provides an effective foundation for organizations to cope with the unexpected in an ever-changing business environment (Parker and Ameen, 2017). Moreover, risk management is the path an enterprise needs to be resilient in today’s dynamic environment (Lisdiono et al., 2022).

In the poultry industry, the relationship between effective leadership and risk management is a pivotal factor in enhancing sector resilience and ensuring long-term sustainability. This industry faces multiple challenges, including fluctuations in feed and product prices, financial crises, outbreaks of diseases such as avian influenza, and climate-related changes affecting productivity. To address these risks effectively, production facilities require competent leadership capable of making swift and well-informed decisions, such as reassessing budgets during feed price surges, adjusting production plans to minimize losses during crises, or diversifying revenue streams by introducing new products like organic eggs or poultry meat. Proactive risk management complements this role by enabling early identification of threats, assessing their impact, implementing preventive measures such as improved ventilation systems and vaccination programs, and quickly redirecting production operations to mitigate potential losses. Integrating strategic leadership with risk management not only ensures continuity of production and reduces economic losses but also strengthens consumer confidence, enhances local food security, and secures the sector’s competitiveness, thereby reinforcing the long-term sustainability of the poultry industry.

Ultimately, it becomes evident that equipping students in poultry science programs with leadership, crisis management, and risk management skills is a critical step in enhancing their professional and academic readiness to face the complex and growing challenges in the sector. By developing these skills, students are empowered to make swift, well-informed decisions and efficiently manage production teams within farms and institutions, while also being capable of early risk detection and effective response to health, economic, or environmental threats through thorough risk assessment and the implementation of well-planned preventive strategies. This preparation further strengthens their ability to innovate and adapt to rapid changes in production markets, supply chains, and climatic conditions, thereby increasing their employability and practical competence. Consequently, fostering leadership and crisis and risk management skills among students not only equips them for the labor market but also contributes to the sustainability of the poultry industry by maintaining production continuity, reducing economic losses, and enhancing organizational resilience, ultimately supporting both local and global food security.

### Strategies for higher education institutions and poultry science programs to prepare students as leaders in risk and crisis management within the poultry industry

Risk management is critically important for both crop farmers and livestock producers. However, there is a significant lack of information on how livestock producers perceive and manage risks. Although existing risk management tools for livestock are effective, livestock producers tend to use them less frequently compared to crop farmers. This may be due to differences in the nature of risks between the two sectors, or because some producers view these tools as insufficient, or they lack the necessary training and motivation to apply them properly. Therefore, it is essential to provide a wider range of structured risk management tools, along with targeted training programs to enhance their effective use. Developing successful educational programs in this field requires a thorough understanding of producers’ needs and interests, which will help in designing tailored tools and programs to support livestock producers in managing risks more efficiently in the future (Hall et al., 2003).

To address the various risks facing the poultry industry, specific strategies can be adopted. For instance, farmers can reduce production risks by investing in advanced technologies, while market risks can be mitigated through securing long-term sales contracts or developing new marketing channels. Financial risks can be managed by diversifying funding sources and implementing careful financial planning. Continuous awareness of laws and regulations is essential to handle legal risks and ensure compliance. Regarding human resource risks, investing in employee training and developing incentive programs enhances workforce performance and resilience (Murrja et al., 2023).

The modern poultry industry operates within a complex production environment characterized by uncertainty and rapid changes, where biological, economic, environmental, geopolitical, and health factors intersect, increasing the likelihood of multifaceted crises. Amid the growing challenges faced by this sector at both local and global levels, there is an urgent need to prepare a new generation of leaders capable of managing risks and crises with high efficiency and effectiveness. Universities play a pivotal role in developing these leaders by adopting advanced educational and training strategies that integrate theoretical knowledge with practical application, while honing the leadership and analytical skills necessary to navigate complex challenges. Consequently, universities can play a vital role in designing specialized programs that prepare students to become pioneers in crisis management, ensuring the sustainability of the poultry sector and contributing to the enhancement of food security and sustainable development through a set of fundamental pillars, which include:

#### Developing a specialized curriculum on crisis and risk management in the poultry industry

Developing a comprehensive academic curriculum focused on crisis and risk management in the poultry industry is a strategic step to enhance students’ preparedness for future challenges in this vital sector. The curriculum aims to provide students with a deep understanding of risk management concepts, emergency response strategies, preventive measures, and practical case studies drawn from the realities of the poultry industry. The curriculum integrates theoretical foundations with applied learning, emphasizing the use of modern technologies and best international practices to ensure the preparation of professionals capable of making sound decisions under crisis conditions. It also seeks to develop leadership skills and the ability to coordinate and collaborate among various stakeholders during emergencies, thereby strengthening the sector’s resilience and sustainability. Through this curriculum, students will not only learn to understand and manage risks effectively but also contribute to building a more resilient poultry sector, prepared to face future challenges and enhance the sustainability and food security of the industry.

#### Developing specialized leadership training programs for students

Specialized leadership training programs for poultry industry students represent a strategic approach to preparing future leaders capable of effectively managing risks and crises. These programs focus on honing leadership skills and enabling rapid, well-informed decision-making during sector-specific crises and risks, such as disease outbreaks, supply chain disruptions, and market fluctuations, while equipping students to strategically plan risk management and ensure production stability.

The programs also include the development of clear emergency response plans with well-defined roles and responsibilities for all stakeholders within the production system, ensuring coordinated and effective action during crises. They emphasize the continuous updating of these plans based on scientific advancements and lessons learned from past experiences, as well as familiarizing students with early warning mechanisms to detect risks before they escalate. In addition, the ultimate goal of this training is to graduate professionals ready to assume leadership roles in the poultry sector, capable of efficiently managing risks and crises, ensuring production continuity under challenging conditions, enhancing sector resilience and long-term sustainability, and contributing to both local and global food security.

#### Promoting the culture of risk management and sustainability

Raising awareness among poultry science students about sustainability and risk management across all levels of the poultry sector to ensure the adoption of responsible production practices that reduce environmental and health risks and enhance systematic crisis management. For example, biosecurity is the first line of defense against biological risks in poultry production, making its rigorous implementation essential. This includes regular disinfection, quarantining new flocks before integration, and monitoring movement of personnel and equipment within farms. Continuous worker training raises awareness of potential risks and prevention methods, reducing human errors that can cause disease spread.

Moreover, integrating technical measures with human expertise creates a safer, more stable production environment and enhances crisis management capacity. Additionally, overreliance on a single source for critical inputs such as feed or vaccines increases vulnerability, especially in developing countries. Therefore, diversifying supply sources and establishing alternative local and regional supply networks reduces the impact of global supply disruptions. Strategic partnerships with suppliers and maintaining reserve stocks ensure supply continuity during emergencies, enhancing sector resilience and reducing financial and production risks from market volatility or natural and political disasters, thereby supporting poultry production sustainability.

#### Enhancing soft skills and effective communication

Training students in poultry science programs to become future leaders in the poultry industry in communication, conflict resolution, and teamwork under crisis conditions is crucial to ensure effective coordination among diverse teams within the sector and to achieve rapid and successful responses in critical situations. From this perspective, poultry science programs should focus on preparing students to become specialists and leaders capable of effectively managing risks and crises through educational and training curricula designed to enhance their theoretical and practical competencies in this field. Providing students with the necessary theoretical knowledge and practical skills to handle various potential disaster and crisis scenarios is considered a cornerstone for the sustainability of the sector.

Furthermore, poultry science programs should aim to refine students’ critical thinking and rapid decision-making skills under pressure, enabling graduates to contribute effectively to emergency planning and the implementation of swift crisis responses. Integrating joint research projects and crisis simulation workshops within these programs, in collaboration with farms and poultry companies, provides practical experience and enhances students’ leadership readiness to face future challenges in the poultry industry.

#### Raising awareness of social responsibility and professional ethics

Risk governance provides a fundamental framework for applying governance principles to the processes of risk identification, assessment, management, and communication through the actions, procedures, traditions, and institutions by which authority is exercised and decisions are made and implemented (SRA, 2015). It underscores the importance of collective decision-making, stakeholder engagement, and the establishment of clear regulatory mechanisms for risk (Thekdi and Aven, 2021). In this context, integrating social responsibility values and professional ethics into leadership training programs in the poultry industry represents a pivotal step toward ensuring effective crisis management. Such integration safeguards consumer health and environmental safety while simultaneously strengthening community trust in poultry products.

Moreover, risk management within the poultry sector encompasses a balanced set of measures and activities designed to capture development opportunities on the one hand, while preventing losses, accidents, and disasters on the other. Its strategies range from risk avoidance, mitigation, and optimization, to transfer, sharing, retention, acceptance, and tolerance. Furthermore, risk management involves policy and risk analysis, resource allocation, trade-off evaluation, goal setting, and performance measurement. In addition, applying these concepts within the poultry industry not only facilitates the management of current crises but also promotes sustainable production, striking a balance between economic growth and safety requirements. Ultimately, this enhances consumer confidence in poultry products and reinforces the industry’s competitive position in both local and global markets.

#### Continuous evaluation and knowledge management

Establishing periodic mechanisms to evaluate the poultry industry's response to past crises and compiling lessons learned into knowledge databases helps new leaders improve risk management strategies and avoid repeated mistakes. Knowledge sharing in turbulent environments enhances new product development and innovation performance. Khvatova et al. (2016) found that information and communication technologies facilitated knowledge sharing and stakeholder collaboration during crises, enabling rapid organizational adaptation to changing contexts. Wang’s (2009) case study revealed that inter-organizational knowledge sharing improves employees’ ability to recognize crisis onset and manage or prevent it. Social activities and direct interactions facilitate collecting, transferring, and applying relevant knowledge to develop effective crisis strategies, enhancing organizational performance in prevention and preparedness phases. During crisis mitigation, documented knowledge and direct employee interactions support knowledge transfer and application to counteract negative impacts and aid recovery (Buhagiar and Anand, 2021).

Moreover, poultry science students should be trained on continuous evaluation and knowledge management through integrated theoretical and practical curricula including case studies and crisis simulation workshops, using knowledge databases to analyze lessons learned. This fosters critical thinking, teamwork, and collaboration with industry experts via lectures and mentorship. Continuous assessment with feedback improves performance, preparing students to become leaders capable of developing effective risk management strategies to sustain the sector.

#### Artificial intelligence applications and modern technologies

Artificial intelligence applications and modern technologies play a fundamental role in improving poultry management by providing accurate, real-time information on flock health, environmental conditions, and market trends. For example, Artificial intelligence -based flock monitoring systems can track growth rates, feed consumption, and detect early signs of disease, allowing preventive measures to be taken before outbreaks occur. Environmental sensing technologies help monitor temperature, humidity, and air quality in poultry houses, ensuring a healthy and stable environment for the birds and reducing the risk of disease or production losses. On the market side, predictive data tools can forecast fluctuations in feed or poultry product prices, enabling farmers to plan financially and minimize economic losses.

Therefore, poultry science programs should provide both theoretical and practical training on these advanced technologies, equipping students with the skills needed to make and analyze data-driven decisions effectively. For instance, students can learn how to use flock health monitoring systems to analyze growth and production data or rely on market forecasting tools to determine optimal times for buying and selling. This training enhances students’ ability to manage risks efficiently, maintain production continuity, ensure supply chain safety, and increase the sector’s resilience against biological and economic crises, ultimately contributing to the long-term sustainability of the poultry industry.

## Conclusion

The modern world has grown increasingly complex due to civilizational and economic transformations, coupled with the pressures of globalization. The real challenge lies not in the crises themselves, but in individuals’ ability to manage them effectively and responsibly. Education serves as a cornerstone in addressing these challenges, extending beyond the mere transmission of academic knowledge to shape character, instill values, and equip learners with the skills needed to face the future with confidence and resilience. In this context, higher education institutions occupy a leading role by providing practical experiences that build self-assurance and enhance students’ capacity for effective crisis management. The poultry sector, in particular, exemplifies an industrial environment where multiple factors converge, making careful crisis and risk management essential.

The poultry industry operates within an increasingly complex and interconnected global environment, where biological, economic, environmental, and social factors intersect to create a wide array of risks and challenges. Effective management of these challenges is critical to ensuring production continuity and sustainability, as well as safeguarding food security at both regional and global levels. This study highlights the urgent need to prepare future leaders in the poultry sector, equipped with the necessary skills to manage risks and crises strategically. Moreover, higher education institutions and poultry science programs can play a pivotal role by offering comprehensive education that integrates theoretical knowledge, practical training, and leadership development. By enhancing students’ abilities in strategic decision-making, problem-solving, and resilience building, academic institutions empower future professionals to anticipate crises, mitigate their impacts, and respond efficiently and effectively.

The study recommends reviewing and updating poultry science programs to incorporate concepts of risk and crisis management, while offering advanced training in collaboration with industry partners and stakeholders. It also emphasizes the importance of recognizing risk management as a core component of academic quality standards to ensure sector sustainability and promote professional and academic excellence. Furthermore, the study provides guidance for policymakers and decision-makers in higher education on preparing students to become strategic leaders capable of managing crises efficiently, making informed decisions, and ensuring the sustainability of the poultry industry in the face of future challenges. Ultimately, investing in the development of such leaders strengthens sector resilience, supports sustainable production systems, and contributes significantly to global food security and long-term societal stability.

## Disclosures

All authors declare that there are no conflicts of interest regarding the publication of this paper.

## Funding

This work was supported by the Deanship of Scientific Research, Vice Presidency for Graduate Studies and Scientific Research, King Faisal University, Saudi Arabia (Grant No. KFU252910).

## CRediT authorship contribution statement

**Farid S. Nassar:** Writing – review & editing, Visualization, Supervision, Software, Resources, Project administration, Methodology, Funding acquisition, Formal analysis, Data curation, Conceptualization. **Ahmed O. Abbas:** Writing – review & editing, Validation, Methodology, Investigation.
